# Introgression of Bacterial Blight Resistance Genes in the Rice Cultivar Ciherang: Response against *Xanthomonas oryzae* pv. *oryzae* in the F_6_ Generation

**DOI:** 10.3390/plants10102048

**Published:** 2021-09-29

**Authors:** Priya Lal Biswas, Ujjal Kumar Nath, Sharmistha Ghosal, Gayatri Goswami, Md. Shalim Uddin, Omar M. Ali, Arafat Abdel Hamed Abdel Latef, Alison M. Laing, Yong-Ming Gao, Akbar Hossain

**Affiliations:** 1 National Key Facility for Crop Gene Resources and Genetic Improvement, Institute of Crop Sciences, Graduate School of Chinese Academy of Agricultural Sciences (GSCAAS), Haidian District, Beijing 100081, China; priyalal.biswas@yahoo.com (P.L.B.); irriygao@126.com (Y.-M.G.); 2 Bangladesh Rice Research Institute (BRRI), Joydevpur, Gazipur 1701, Bangladesh; sharmi.brri@gmail.com; 3 Department of Genetics and Plant Breeding, Bangladesh Agricultural University, Mymensingh 2202, Bangladesh; ujjalnath@gmail.com; 4 Department of Genetics and Plant Breeding, Patuakhali Science and Technology University, Patuakhali 8600, Bangladesh; gayatri_bau@yahoo.com; 5 Bangladesh Agricultural Research Institute, Gazipur 1701, Bangladesh; shalimuddin40@gmail.com; 6 Department of Chemistry, Turabah University College, Turabah Branch, Taif University, P.O. Box 11099, Taif 21944, Saudi Arabia; 7 Botany and Microbiology Department, Faculty of Science, South Valley University, Qena 83523, Egypt; moawad76@gmail.com; 8 CSIRO Agriculture & Food, St. Lucia, QLD 4067, Australia; alison.laing@csiro.au; 9 Department of Agronomy, Bangladesh Wheat and Maize Research Institute, Dinajpur 5200, Bangladesh

**Keywords:** rice, bacterial blight, *Xanthomonas oryzae* pv. *oryzae*, resistance genes, pyramiding, marker-assisted selection

## Abstract

Bacterial blight (BB) is caused by *Xanthomonas oryzae* pv. *oryzae* and is one of the most important diseases in rice. It results in significantly reduced productivity throughout all rice-growing regions of the world. Four BB resistance genes have been reported; however, introgression of a single gene into rice has not been able to sufficiently protect rice against BB infection. Pyramiding of effective BB resistance genes (i.e., *Xa* genes) into background varieties is a potential approach to controlling BB infection. In this study, combinations of four BB resistance genes, *Xa4*, *xa5*, *xa13*, and *Xa21*, were pyramided into populations. The populations were derived from crossing Ciherang (a widespread Indonesian rice variety) with IRBB60 (resistance to BB). Promising recombinants from the F_6_ generation were identified by scoring the phenotype against three virulent bacterial strains, C5, P6, and V, which cause widespread BB infection in most rice-growing countries. Pyramiding of genes for BB resistance in 265 recombinant introgressed lines (RILs) were confirmed through marker-assisted selection (MAS) of the F_5_ and F_6_ generations using gene-specific primers. Of these 265 RILs, 11, 34 and 45 lines had four, three, or two BB resistance genes, respectively. The RILs had pyramiding of two or three resistance genes, with the *Xa4* resistance gene showing broad spectrum resistance against *Xoo* races with higher agronomic performance compared to their donor and recipients parents. The developed BB-resistant RILs have high yield potential to be further developed for cultivation or as sources of BB resistance donor material for varietal improvement in other rice lines.

## 1. Introduction

Globally, rice accounts for more than 21% of total food intake; it also provides up to two-thirds of the total calories consumed by more than two billion people across Asia, Africa, and Latin America [1,2,3,4]. It is estimated that rice production must increase by 0.6–0.9% annually until 2050 in order to meet the demand of an increasing world population [5]. However, rice yields have plateaued due to biotic and abiotic stresses [6]. Among these, bacterial blight (BB), which is caused by *Xanthomonas oryzae* pv. *Oryzae* (*Xoo*), is one of the most destructive diseases, limiting rice production around the world. BB was first identified in Japan at the beginning of the twentieth century [7]. It can cause damage at any stage of the rice-growing cycle. BB causes yield losses generally ranging between 10 and 30%, but which can be as high as 80%, depending on the location, season, weather, crop growth stage, and cultivar [8,9,10,11]. There are no chemicals or management practices known to reduce the severity of BB [12]. The development of a BB-resistant rice cultivar through a gene introgression breeding program is critically important [13,14,15].

To date, at least 45 genes across 10 of 12 rice chromosomes have been identified which confer resistance to various strains of *Xoo* [16,17,18,19]. Among these resistance genes, 17 (*xa5, xa8, xa13, xa15, xa19, xa20, xa24, xa25, xa26, xa28, xa31, xa32, xa33, xa34, xa41, xa42* and *xa44*) are recessive, while the remaining 28 are dominant [20,21,22]. All the genes are in the series from *Xa1* to *Xa45* [17]. Out of the 45 resistance genes, nine (*Xa1, Xa3/xa26*, *xa5, Xa10, xa13, Xa21, Xa23, xa25* and *Xa27*) have been cloned and twelve (*Xa2, Xa4, Xa7, Xa22, Xa30, xa31, xa33*, *xa34, Xa38, Xa39*, *Xa40* and *Xa42*) have been physically mapped [20,21,22,23,24,25,26,27,28,29]. Some of these resistance genes have been successfully incorporated into rice cultivars that are now extensively cultivated in many rice-growing countries [13,15,30]. Most of the resistance genes follow the classic gene-for-gene concept against the race-specific interaction between rice and *Xoo* [31]. Most of the genes show resistance in all growing stages of rice, while only a few resistance genes, such as *xa13*, show resistance to *Xoo* only at mature plant stages, and *xa5* and *Xa4* show a broad spectrum of resistance to *Xoo* isolates [32,33,34]. The gene *Xa21,* which is found in the wild rice species *O. longistaminata,* shows resistance against BB at the seedling stage [35] and has the potential to be highly effective against BB in South and South-East Asia [32,33,34,36].

Rice lines with multiple BB resistance genes have a wider and more durable level of resistance than those lines that have only a single BB resistance gene [15,26,37,38,39]. Large-scale, long-term cultivation of rice cultivars carrying a single BB resistance gene may not be sufficient to beat the BB pathogen and resistance will not persist in a long time. Conventional breeding approaches are not efficient enough for the rapid identification of BB resistance genes [40,41,42].

The use of molecular markers to select a particular trait has numerous advantages over the morphological markers of conventional plant breeding [14,39]. We examined the potential for marker-assisted selection to identify rice lines with multiple BB resistance genes (i.e., *Xa* genes). It is possible to develop closely linked molecular markers for each of the resistance genes within the plants, for their easy identification [15,30,43]. Three BB resistance genes (*xa5*, *xa13,* and *Xa21*) were incorporated in the cultivar PR106 through marker-assisted selection (MAS), showing a broad spectrum of resistance against 17 *Xoo* isolates under field conditions [15]. This study used phenotypic and molecular markers to identify the BB resistance genes *Xa4*, *xa5*, *xa13*, and *Xa21* in recombinant inbred lines (RILs) derived from a cross between the rice varieties ‘Ciherang’ and ‘IRBB60’.

## 2. Results

### 2.1. Response of RILs toward BB Races

**Figure 1:**
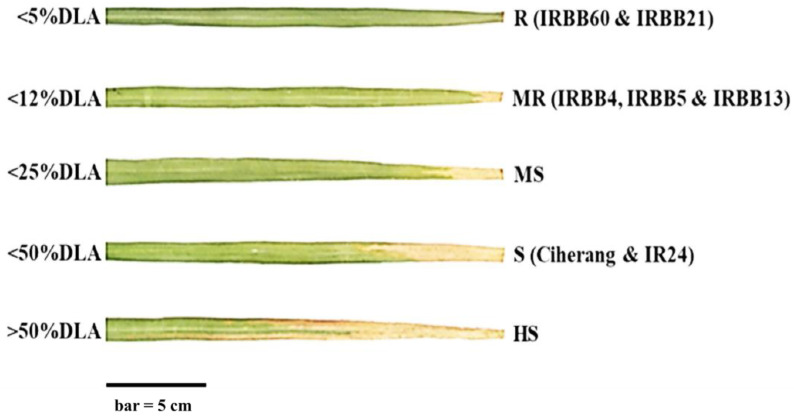
Disease scoring of infected leaves fourteen days after inoculation with three BB races. DLA—diseased leaf area, R—resistant, MR—moderately resistant, MS—moderately susceptible, S—susceptible, HS—highly susceptible.

**Figure 2:**
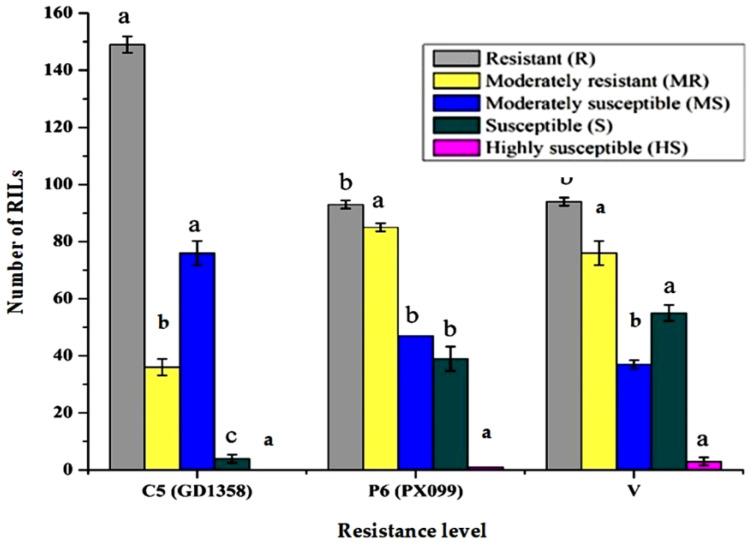
Response of plants in RIL to inoculation of fungus. Data are the average of 3 replications with disease scored from 5 leaves in each replication. Mean separation was performed by Tukey test (*p* ≤ 0.05). The notation indicates resistant (R), moderately resistant (MR), moderately susceptible (MS), susceptible (S) and highly susceptible (HS).

To assess the level of virulence of the isolates, the leaf area progressed with BB and the percentage of resistance in RILs were examined (Figure 1). Of the three races (i.e., C5 (GD1358), V, and P6 (PX099)), P6 and V were more virulent than C5. Of the total of 265 RILs, only 93 and 94 showed resistance against the P6 and V races, respectively, while 149 RILs were resistant against C5 (Figure 2).

Eighty-five RILs (32%) were moderately resistant against the P6 race, 76 (29%) moderately resistant against V, while 36 RILs (14%) had moderately resistant against C5. In contrast, the number of susceptible or moderately susceptible RILs against each of the races was 92 (35%) for V, 86 (32%) for P6, and 80 (30%) for C5. Both the susceptible check (IR24) and the recipient parent (Ciherang) showed susceptibility against all the tested races. The resistance checks (IRBB5, IRBB13, and IRBB21) and their donor parent (IRBB60) showed resistance or moderately resistant against all three races, and the *Xa4* resistance gene bearing the IRBB4 genotype showed resistance against the C5 and P6 races, but was moderately susceptible to V, indicating race-specific resistance of the *Xa4* gene. Of the 265 RILs, only three lines were highly susceptible to race V. One was highly susceptible to P6, and no lines were highly susceptible to C5. We identified a higher amount of RILs resistant to C5 than in the other two races (Figure 2), indicating that C5 was comparatively weaker than the V and P6 races.

Two hundred and seventeen RILs (82% of all RILs) were resistant to moderately resistant against single to multiple BB races. Against the three tested races, 55 RILs were resistant and a further eight were moderately resistant (Table 1).

### 2.2. Molecular Marker Analysis for Characterizing the RILs

A total of 265 RILs were screened by PCR using molecular markers to identify the BB resistance genes (*Xa4*, *xa5*, *xa13*, and *Xa21*). These RILs had a combination of single, double, triple and quadruple resistance genes in different combination (Table 2). For all the tested markers, the recipient parent ‘Ciherang’ produced a PCR amplicon identical to that of the susceptibility check IR24, while the BB resistant donor parent ‘IRBB60’ generated a PCR amplicon identical to that of the four resistance checks.

**Figure 3:**
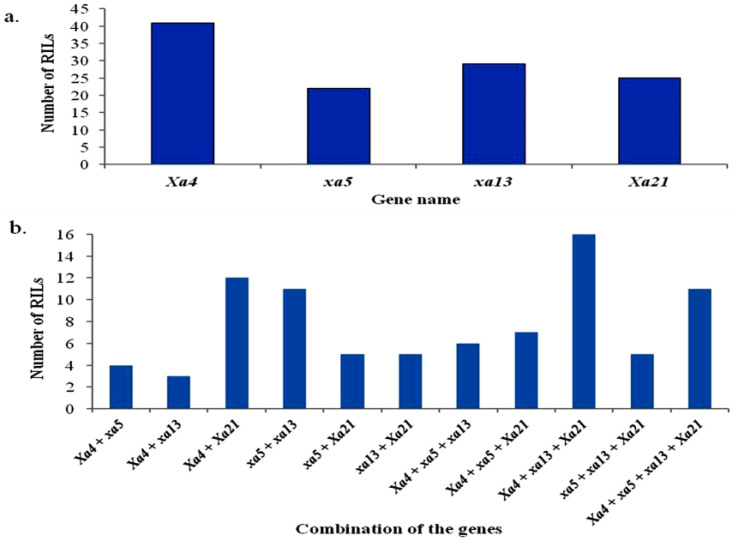
Molecular characterization of the RILs using gene-based and gene-linked markers: (**a**) number of RILs with a single resistance gene, (**b**) the RILs which possessed more than one resistance gene in combination.

Overall, 217 RILs were phenotypically identified as resistant against the three tested races. Of these, 203 RILs had at least one marker amplified by the PCR: a single marker was amplified in 118 RILs and in the remaining 85 RILs, multiple markers were amplified. PCR amplicons were found in 41 RILs for the *Xa4* gene, in 23 RILs for the *xa5* gene, in 29 RILs for the *xa13* gene, and in 25 RILs for the *Xa21* gene (Figure 3a). The PCR results identified 100 RILs that carried the *Xa4* gene, either alone or in combination with other genes (Figure 3b). Of these, 41 RILs carried only the *Xa4* gene, while 19, 29 and 11 RILs amplified the *Xa4* gene in combination with one, two or three other resistance genes, respectively (Figure 3b). Seventy-two RILs produced PCR amplicons of the *xa5* gene; of these, 23 carried only the *xa5* gene, and 20 and 29 RILs amplified the *xa5* gene in combination with one or more than one other gene, respectively. Eighty-six RILs carried the *xa13* gene both in solely or in combination with other loci; of these, 29 RILs carried only the xa13 gene and the remaining 57 RILs had amplicons with two or more genes. In terms of the *Xa21* gene, 25 RILs carried *Xa21* alone and 61 RILs had amplicons with two or more genes (Figure 3b). The RILs that carried dominant genes generally combined with other dominant genes, while those with recessive genes combined with other recessive-gene RILs.

**Figure 4:**
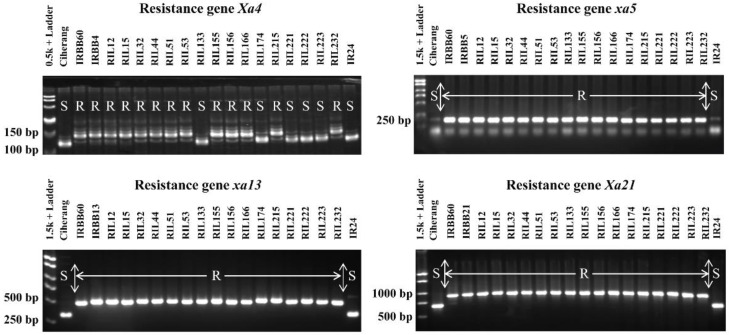
Molecular markers analysis of selected RILs along with their parent lines and resistant and susceptible checks, showing the homozygous condition of the BB resistance genes *Xa4*, *xa5*, *xa13*, and *Xa21*. R = resistant; S = susceptible.

A total of 85 pyramided lines with multiple resistance genes was identified from 203 RILs with R genes. Of these, PCR amplification identified 40, 34, and 11 RILs with two, three, or four resistance genes, respectively. Nineteen of the 85 pyramided lines carried one or more heterozygous alleles of the BB resistance gene(s). These pyramided RILs were evaluated in a pedigree nursery up to the F_7_ generation, and the resistance reaction against BB races was observed. The pyramided RILs that contained four specific resistance genes are shown in Figure 4 along with their parent lines and the resistance and susceptibility checks.

### 2.3. Agronomic Performance of Pyramided Lines

The 85 pyramided RILs previously identified were categorized into 11 groups (G1 to G11) on the basis of the number and combination of the resistance genes (Table 3). These groups were evaluated under field conditions in Beijing to assess their agronomic performance.

There were significant differences between the pyramiding RIL groups in terms of plant height, panicle length, 1000-grain weight, spikelets per panicle, and the length-to-width ratio of the spikelet (Table 4).

RILs were grouped based on the number and combination of different resistance genes present in the RIL genotypes. Most of the selected RILs were combinations of the IRBB60 and Ciherang gene pools, which showed positive resistance against *Xoo* races. In comparison with the donor and recipient parents, the RILs showed better agronomic performance. RILs contained the *Xa4* resistance gene in combination with other any of the resistance genes showed higher agronomic performance for all the studied traits. These data indicates that RILs with three to four introgressed resistance genes had no adverse effect on the agronomic traits. Although one or two resistance genes in combination with the dominant resistance gene *Xa4* were sufficient to resistance response against all three tested virulent *Xoo* races.

### 2.4. Associations between the Number and Combinations of Pyramided Resistance Genes and Disease Reaction against Different BB Races

A principal component analysis of the reaction in different BB races in the RILs identified three principal components (PCs) with eigenvalues near to unity (Table 5). The first three PCs together explained 96.9% of the total variance in BB disease reaction in the RILs: 65.8% of the variance was explained by PC1, 21.3% by PC2 and 9.7% by PC3 (Table 5).

**Figure 5:**
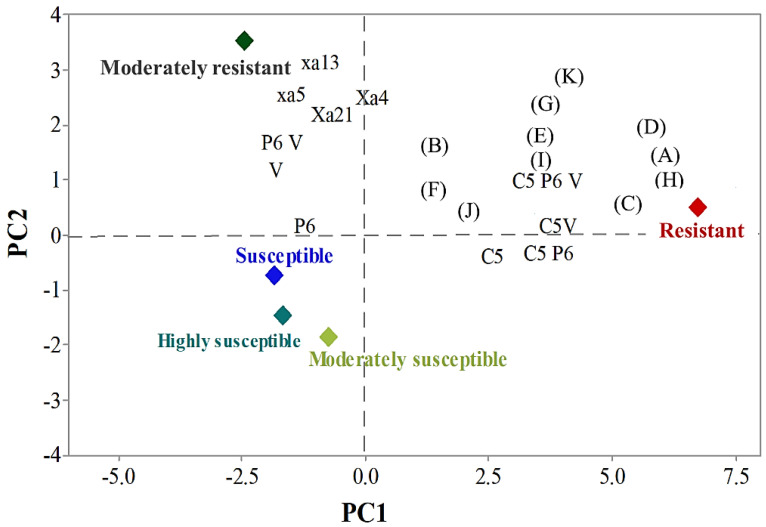
Distribution of RILs in terms of resistant, moderately resistant, moderately susceptible, susceptible, and highly susceptible groups, with their BB resistance genes individually or in pyramided conditions, and the disease reaction against individual or combinations of BB races. The combinations of the four BB resistance genes in the figure are: A: *Xa4 + xa5*; B: *Xa4 + xa13*; C: *Xa4 + Xa21*; D: *xa5 + xa13*; E: *xa5 + Xa21*; F: *xa13 + Xa21*; G: *Xa4 + xa5 + xa13*; H: *Xa4 + xa5 + Xa21*; I: *Xa4 + xa13 + Xa21*; J: *xa5 + xa13 + Xa21* and K: *Xa4 + xa5 + xa13 + Xa21*.

The variation accounted for by PC1 was a result of the higher positive coefficients of the resistance reactions of the combinations of the pyramided genes and the strength of the C5 race in combination with the P6 and V races, compared to the high negative coefficients of the individual resistance genes *xa5*, *xa13*, *Xa4,* and *Xa21*, as well as the weaker disease reaction in RILs in the P6 and V races (Figure 5).

PC1 clearly distinguished between resistant and susceptible RILs (although the identification of moderately resistant RILs was less distinct). This clear distinction corresponded to strong disease-resistance reactions in the pyramided genes in the RILs, either against strong virulent individual races or in a combination of strong and weaker races. Moderately resistant RILs had at least one resistance gene and were moderately resistant against the weaker BB races; therefore these RILs were classified as ‘susceptible’ (Figure 5). These results were applied to the selection of resistant RILs to create the next generation, by excluding those with only moderately resistant.

PC2 divided RILs into resistant and susceptible groups, along with their respective resistant individual or pyramided gene(s) (Figure 5). The susceptible group included those RILs which had no resistance gene; these RILs had mean PC scores of +7.1 and −2.1 from the resistant and susceptible parents, IRBB60 and Ciherang, respectively.

## 3. Discussion

More than half of the global population eats rice to meet their daily dietary requirements. The demand for rice production is increasing, and many studies have reported that global rice production needs to double by 2050 to meet this growing demand [44]. Additionally, there are many biotic and abiotic stresses which affect both the yield quantity and quality of rice crops. To address these constraints and to increase rice production, it is necessary to develop high-yielding cultivars enriched with disease-resistance genes. The development of rice varieties with broad-spectrum resistance against bacterial blight (BB) (caused by the *Xoo* bacteria) is hugely challenging due to the presence of several genetically distinct virulent *Xoo* strains in different rice growing locations in the world [45]. There is little published literature on the development of multiple-race-resistant cultivars. To improve the sustainable cultivation of rice we have planned and executed the introgression of four BB (*Xa4*, *xa5*, *xa13*, and *Xa21*) resistance genes/QTLs into a Ciherang × IRBB60 cross in order to achieve multiple-BB race resistance in rice. We observed distinct polymorphism with four co-dominant markers linked to the BB resistance genes from the Ciherang (recipient) parent and IRBB60 (donor) parent material (Figure 4).

The main objective of this study was to identify and successfully demonstrate the function of the four BB resistance genes (*Xa4*, *xa5*, *xa13,* and *Xa21*) in the RILs of the F_6_ generation. These four target BB resistance genes were identified through bioassays against three BB races (C5, P6, and V) and an effective foreground selection was undertaken using gene-specific markers in 265 RILs in two consecutive generations (F_5_ and F_6_). These markers were MP, xa5, xa13, and pTA248 for the *Xa4*, xa5, xa13, and *Xa21* genes, respectively. PCR products showed approximately 140 bp band for the *Xa4* gene [46,47], 198 bp band for the *xa5* gene, 500 bp for the *xa13* gene [48], and 980 bp for the *Xa21* gene [46,49,50]; these were confirmed by the resistance checks IRBB4, IRBB5, IRBB13 and IRBB21 (Figure 4).

Of 265 lines, 203 had one or more of the targeted BB resistance genes in different combinations (Figure 3), and 40, 34 and 11 lines had two, three, or four BB resistance genes. These recombinant lines exhibited a high level of resistance against three virulent BB isolates, which correlates with the results of [51]. Mundt [52] also reported that the effectiveness of a combination two or more genes is higher than that of a single gene in defeating simultaneous pathogen mutations for virulence, therefore assembling several resistance genes into a host plant is a viable and practical strategy.

We observed inheritance of some unfavorable characteristics (i.e., biomass growth and grain weight) along with some favorable traits while pyramiding genes/QTLs from the parent IRBB60 variety. A “pull” of undesirable genes from the parent occurred when the *Xa21*, *xa13*, and *xa5* genes were introgressed from the SS1113 variety [30]. Ramalingam et al. [53] assessed the homozygous improved pyramiding lines (BC3F3 generation) that harbored the *xa5* + *xa13* + *Xa21* + *Pi54* + *qSBR7-1* + *qSBR11-1* + *qSBR11-2* genes in terms of physical resistance under greenhouse conditions and suggested that pyramiding three BB resistance genes resulted in higher resistance levels than the lines with only one or two BB resistance genes.

**Figure 6:**
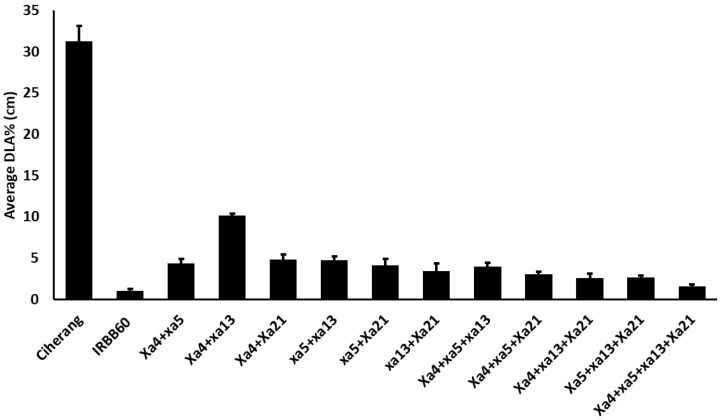
Percentage of disease infection of the 11 groups of pyramided lines with different combinations of genes.

In our bioassay study, 85 pyramided lines which carried at least two BB resistance genes showed a high level of resistance (Figure 3 and Figure 4). Eleven pyramided lines (RIL 12, RIL 15, RIL 32, RIL 44, RIL 51, RIL 53, RIL 155, RIL 156, RIL 166, RIL 215 and RIL 232), all of which had *Xa4 + xa5 + xa13 + Xa21* in combination, had higher resistance performance than the RILS with combinations of other genes (Figure 4) [51,54,55,56,57,58]. These 11 RILs had an average of 1.57 cm of diseased leaf area infected with the three BB races (Figure 6), which is similar to results from other reports [41,47,51,57,59,60]. The enhanced resistance due to the combination of two or more genes compared to the resistance from a single gene is known as synergistic action, or quantitative complementation [61]. This result is also further confirmed and explained by our PC analysis.

The pyramided lines were categorized into 11 groups determined by the number and combinations of genes. These pyramided RILs were significantly different in terms of plant height, panicle length, 1000-grain weight, and the spikelet length-to-width ratio, indicating that these pyramided lines have diverse yield potentiality. The field evaluation of improved pyramided lines of the F_7_ generation demonstrated that selected lines had equivalent yield, agro-morphological, and quality traits and equivalent pyramided genes for BB. This higher level of resistance to BB disease observed in different races without yield penalty is a positive outcome from our approach of integrated genotypic and phenotypic selection methods.

## 4. Materials and Methods

### 4.1. Plant Materials

**Figure 7:**
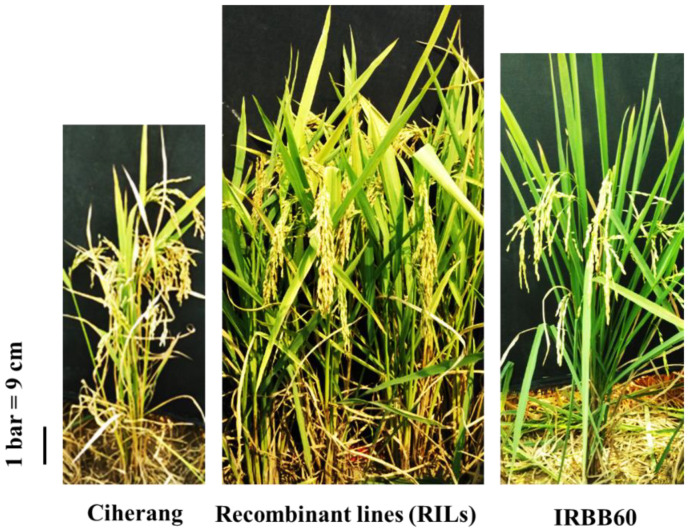
Morphological features of the recipient (Ciherang), donor (IRBB60) parents and their recombinant lines (RILs).

**Figure 8:**
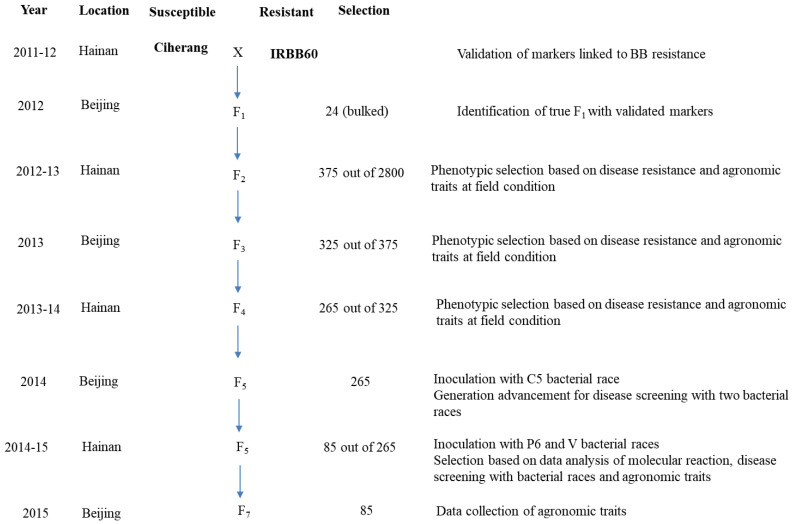
Rice breeding flowchart to illustrate the selection work of each generation of the pyramiding lines against different BB races.

In this study, we used 265 RILs at the F_6_ generation of the ‘Ciherang × IRBB60’ cross, which were recombinant of recipient and donor parents, four gene-specific BB resistant cultivars (viz. IRBB4, IRBB5, IRBB13, IRBB21) and one BB susceptible line (IR24) (Figure 7). Among these genotypes, the recipient parent ‘Ciherang’, *Oryza sativa* ssp. *indica,* is a variety popular in Indonesia which is susceptible to BB, and the donor parent ‘IRBB60’ was developed by the International Rice Research Institute (IRRI) through the pyramiding of four BB resistance genes (*Xa4*, *xa5*, *xa13,* and *Xa21*) into the existing IR24 variety. The plant materials were evaluated up to the F_7_ generation for agronomic traits and the segregation of the pyramided BB resistance genes was followed by molecular markers. A rice breeding flowchart is presented in Figure 8 to illustrate the selection work of each generation of the pyramiding rice lines against three BB races.

### 4.2. Preparation of Inocula to Infect Rice Plants

Three BB races—C5 (GD1358) and V, which are newly virulent strains in China [62], and P6 (PX099), which contained TALE PthXo7 [63] collected from the Philippines—were grown in Wakimoto semi-solid medium (potato 300 g, sucrose 20 g, Na_2_HPO_4_·2H_2_O_2_ 2 g, Ca(NO_3_)_2_·4H_2_O 0.5 g, agar 25 g,) per liter at 25 °C for 72 h and preserved at 4 °C following the standard methodology [64,65,66]. A single colony was further sub-cultured in Wakimoto liquid medium with agitation at room temperature for 72 h and the cell suspension diluted to 10^8^ cells per milliliter of distilled water, which was confirmed by measurement using a spectrophotometer (BOECO MODELS S-200 VIS & S-220 UV/VIS, Hamburg, Germany) with A_600_ OD. This sub-culture was used to inoculate the F_7_ rice plants grown in the field.

### 4.3. Inoculation of Rice Plants with BB Races/Isolates

Seeds of the RILs, their parent varieties, and the check varieties were sown in a 50 × 50 cm seedbed. After 22 days, rice seedlings were manually transplanted into experimental plots in Hainan (18.30 N, 109.30 E). Each plot consisted of two rows with 10 plants per row; spacing was 20 cm row-to-row and 17.5 plant-to-plant. The field experiment was conducted using a randomized complete block design with three replicates. The F_5_ generation of all varieties and RILs were inoculated with the C5 bacterial race at the reproductive stage (i.e., onset of heading) by clipping 2–3 cm from the tip of the flag leaves and removing all other leaves [67]. In each plot, four hills per row were randomly selected and inoculated.

Subsequently, the F_6_ generation of RILs, parents, and check varieties were moved to Beijing (40.20 N, 116.20 E) and inoculated with the V and P6 races. To do this, four hills were randomly selected from the first row of each plot and inoculated with the P6 race; a further four hills were inoculated with the V race. The middle two hills in each row were left without inoculation to avoid contamination of the bacterial races.

### 4.4. Assessment of Disease Response and Scoring

Disease scoring was conducted two weeks after inoculation, following the protocol recommended by the international rice research institute, IRRI [68]. The percentage of diseased leaf area (DLA) was calculated as the lesion length (LL) per total leaf length (TLL) × 100. DLA was categorized as resistant (R) if DLA was 5% or less; as moderately resistant (MR) if DLA was between 6and 12%; as moderately susceptible (MS) if DLA was between 13 and 25%; as susceptible (S) if DLA was between 26 and 50%; and as highly susceptible (HS) if DLA was greater than 50% [68].

### 4.5. DNA Isolation and PCR Analysis

DNA was extracted from leaf samples of four week old seedlings using the CTAB method [69]. The gene-specific and gene-linked markers MP1, MP2, *xa5*, *xa13* prom, and pTA248, which are linked to genes *Xa4*, *xa5*, *xa13*, and *Xa21*, were synthesized by the Open Lab of the Chinese Academy of Agricultural Sciences (Table 6) and used to confirm the presence of resistance genes [70]. The polymerase chain reaction (PCR) was performed using 25 µL mixture, which contained 1 µL of 50 ng DNA, 1 µL of 5 µM of each forward and reverse primers, 0.5 µL of 5.0 mM dNTPs, 2.5 µL of 10× PCR buffer (500 mM KCL, 100 mM Tris-HCl pH 8.4, 15 mM MgCl_2_, 0.1% gelatin), 1.8 µL of 25 mM MgCl_2_, 1.0~0.75 units/µL Taq polymerase, and 16.2 µL sterile distilled H_2_O, for the MP1, MP2, *xa13* prom and pTA248 markers.

A reaction mixture of 20 µL, consisting of 5 µL DNA (50 ng/µL), 2 µL of 5 µM of each forward and reverse primers, 0.4 µL of 10 mM dNTPs, 2 µL of 10× PCR buffer, 1.6 µL of 25 mM MgCl_2_, 0.4 of 5 units/µL Taq polymerase and 6.6 µL sterile distilled H_2_O, was used for the *xa5* marker. For the MP1 and MP2 markers, the PCR profile was followed with the initial denaturation at 94 °C for 4 min followed by 35 cycles of denaturation for 1 min at 94 °C, annealing for 1 min at 56 °C and extension for 2 min at 72 °C, with the final extension for 8 min at 72 °C. The PCR profile was followed with the initial denaturation for 5 min at 94 °C followed by 35 cycles of denaturation for 30 s at 94 °C and annealing for 30 s for the *xa13* gene; at 1.4 min for the pTA248 markers targeted to the *Xa21* gene at 55 °C with a final extension for 10 min at 72 °C. For the for *xa5* gene, the PCR was followed with an initial denaturation for 5 min at 94 °C, followed by 35 cycles of denaturation for 1 min at 94 °C, annealing for 1 min at 68 °C and extension for 1 min at 72 °C, with a final elongation for 4 min at 72 °C.

PCR amplicons were mixed with 5 µL loading dye (loading dye:SYBR = 3:1) and visualized under UV-light after electrophoresis with an 8% polyacrylamide gel for *Xa4*, with 1.5% agarose gel for the *xa13* gene, and a 2% agarose gel for the *Xa21* and *xa5* genes.

### 4.6. Data Collection and Analysis

Five plants were sampled from each pyramided line at maturity stage, from which yield and yield-contributing traits were measured. These traits were the number of days until 50% heading was achieved, plant height, the number of effective tillers per hill, panicle length, the number of spikelets per panicle, the percentage of spikelets which were fertile, the spikelet length-to-width ratio, the 1000-grain weight, and the grain yield per plant. Data were analyzed using Microsoft Office Excel 2007 and Statistix 10 software (http://www.biosci.global/softwar-en/genstat/, accessed on 15 July 2021).

## 5. Conclusions

This study aimed to identify bacterial blight-resistant pyramided rice lines in RILs of the F_6_ generation derived from a cross between the Ciherang and IRBB60 parent varieties. Eighty-five pyramided lines (carrying two to four combinations of the BB resistance genes *Xa4*, *xa5*, *xa13*, and *Xa21*) were analyzed using phenotypic and MAS techniques. We observed improved agronomic performance of the pyramided lines compared to that of their parent lines or of the check varieties. These pyramided rice lines have potential to be used as agronomic cultivars and/or potential source for increased BB resistance in future breeding programs.

## Figures and Tables

**Table 1:** Phenotypic disease response of 265 RILs against different BB races of rice considering single and multiple races together.

Races	Phenotypes			
	R	MR	MS	S
C5	38	4	21	0
P6	8	17	0	0
V	7	6	0	0
C5 + P6	27	5	10	0
C5 + V	29	7	4	0
P6 + V	3	3	0	0
C5 + P6 + V	55	8	11	2
Total	217		48	

**Table 2:** Phenotypic and genotypic status of 265 RILs assessed by bioassay against three BB races screened with four markers.

Phenotypic Status	Genic Status *	RILs with Different Genes			
		*Xa4*	*xa5*	*xa13*	*Xa21*
	Single locus	41	23	29	25
Resistant	Double loci	19	20	19	22
	Triple loci	29	18	27	28
	Quadruple loci	11	11	11	11
Susceptible	No amplification	165	193	179	179
Total		265	265	265	265

* Single locus: presence of any one gene out of targeted four pyramiding loci; Double loci: presence of any 2 genes out of targeted four pyramiding loci; Triple loci: presence of any 3 genes out of targeted four pyramiding loci; and Quadruple loci: presence of any 4 genes out of targeted four pyramiding loci.

**Table 3:** Grouping of the pyramided RILs with more than one resistance gene.

Group	R Genes	Number of RILs with Tag Number within Parenthesis
G1	*Xa4* + *xa5*	4 (68, 78, 163, 219)
G2	*Xa4* + *xa13*	3 (107, 121, 128)
G3	*Xa4* + *Xa21*	12 (16, 28, 63, 66, 69, 91, 126, 164, 180, 189, 212, 255)
G4	*xa5 + xa13*	11 (39, 43, 52, 61, 71, 117, 144, 182, 202, 210, 253)
G5	*xa5* + *Xa21*	5 (109, 134, 143, 213, 252)
G6	*xa13* + *Xa21*	5 (11, 77, 191, 211, 248)
G7	*Xa4* + *xa5* + *xa13*	6 (47, 58, 64, 206, 235, 262)
G8	*Xa4* + *xa5* + *Xa21*	7 (7, 34, 74, 94, 186, 207, 199)
G9	*Xa4* + *xa13* + *Xa21*	16 (3, 31, 35, 40, 42, 49, 57, 79, 118, 122, 127, 141, 161, 162, 194, 247)
G10	*xa5* + *xa13* + *Xa21*	5 (133, 174, 221, 222, 223)
G11	*Xa4* + *xa5* + *xa13* + *Xa21*	11 (12, 15, 32, 44, 51, 53, 155, 156, 166, 215, 232)

**Table 4:** Agronomic performance of the 85 BB resistance RILs relative to their donor and recipient parents, data represent average of 3 replications and number of RILs in a particular group.

Genotype	HD	PH	TN	PL	S/P	SF%	L/W	TGW	Y
Ciherang	108.00i	83.33f	9.67ab	20.09i	114.33f	79.83c–e	3.25b–d	24.15c–f	16.10i
IRBB60	117.00c–e	82.13f	9.27a–c	21.85h	125.27ef	86.57ab	2.76e	23.51e–g	17.24gh
G1	119.17a–c	90.57de	7.00gh	23.75d–f	189.30ab	77.32e	3.41ab	23.41f–h	19.38bc
G2	120.78a	90.42de	8.01c–g	23.06fg	117.70ef	83.49a–d	3.42ab	23.56e–g	22.64a
G3	120.44ab	94.58cd	6.85gh	24.22c–e	163.05b–d	78.89de	3.47a	25.29ab	19.92b
G4	114.91e–g	100.82ab	8.66b–f	25.26a	186.13ab	85.48ab	3.43a	24.48b–f	18.56c–e
G5	116.47d–f	96.12b–d	7.77d–g	24.52a–d	193.21a	85.45ab	3.45a	25.27a–c	18.68fg
G6	116.60de	95.37b–d	8.83b–e	24.20c–e	172.03ab	79.71de	3.49a	23.87d–f	16.63hi
G7	118.11cd	103.59a	7.91c–g	25.31a	187.23ab	83.55a–d	3.43a	25.15a–c	18.16ef
G8	113.10gh	99.91a–c	7.94c–g	23.56ef	190.78a	81.54b–e	3. 41ab	24.58a–e	19.08cd
G9	116.67de	100.18a–c	8.85b–e	25.04ab	191.44a	85.21a–c	3.38a–c	24.72a–d	19.29bc
G10	116.80de	96.33b–d	10.36a	24.72a–c	142.67de	87.46a	3.53a	25.64a	18.29d–f
G11	116.55d–f	101.36ab	9.16a–d	25.20ab	171.65a–c	88.26a	3.42ab	24.76a–d	18.57c–e
IRBB4	114.33fg	88.00ef	7.67e–h	23.21fg	140.00df	79.67de	3.17d	22.36hi	18.45d–f
IRBB5	115.67ef	85.44ef	7.00gh	23.32fg	128.33ef	83.28a–d	3.08d	22.13i	17.63fg
IRBB13	111.00h	86.00ef	6.67gh	23.50e–f	128.67ef	83.00a–d	3.19d	22.70g–i	16.89g–i
IRBB21	118.33b–d	86.56ef	7.33f–h	24.45b–d	120.00ef	83.53a–d	3.20cd	22.60g–i	18.26d–f
IR24	115.33ef	99.00a–c	6.33h	22.58gh	144.33c–e	69.67f	3.08d	22.28hi	16.11i
*p* (≤0.05)	0.01	0.01	0.01	0.01	0.01	0.01	0.01	0.01	0.01
St dev (±)	0.72	2.00	0.46	0.26	9.00	1.78	0.06	0.37	0.27

HD = days to 50% heading, PH = plant height (cm), TN = number of effective tillers, PL = panicle length (cm), S/P = spikelets per panicle, SF% = percentage of spikelet fertility, L/W = length-to-width spikelet ratio, TGW = 1000-grain weight (g), and Y = yield per plant (g).

**Table 5:** Component loadings and mean principal component (PC) scores, showing (1) the association between resistant, moderately resistant, moderately susceptible, susceptible, and highly susceptible groups, along with their BB resistance gene(s) individually or in pyramiding conditions; and (2) the disease reaction in BB races (individually or in combinations) as determined using the principal component analysis. The combinations of resistance genes are grouped alphabetically for easier representation in Figure 5.

Variables	PC1	PC2	PC3
C5	0.189	−0.145	0.11
P6	−0.084	−0.041	0.644
V	−0.123	0.21	0.514
C5 P6	0.234	−0.096	0.201
C5V	0.242	−0.063	0.191
P6 V	−0.117	0.283	0.436
C5 P6 V	0.26	0.021	0.091
*Xa4*	0.009	0.454	−0.09
*xa5*	−0.06	0.44	−0.092
*xa13*	−0.047	0.445	−0.092
*Xa21*	−0.036	0.448	−0.092
*Xa4 + xa5* (A)	0.259	0.061	0.006
*Xa4 + xa13* (B)	0.259	0.061	0.006
*Xa4 + Xa21* (C)	0.259	0.061	0.006
*xa5 + xa13* (D)	0.259	0.061	0.006
*xa5 + Xa21* (E)	0.259	0.061	0.006
*xa13 + Xa21* (F)	0.259	0.061	0.006
*Xa4 + xa5 + xa13* (G)	0.259	0.061	0.006
*Xa4 + xa5 + Xa21* (H)	0.259	0.061	0.006
*Xa4 + xa13 + Xa21* (I)	0.259	0.061	0.006
*xa5 + xa13 + Xa21* (J)	0.259	0.061	0.006
*Xa4 + xa5 + xa13 + Xa21* (K)	0.259	0.061	0.006
Eigenvalue	14.474	4.689	2.145
Proportion	0.658	0.213	0.097
Cumulative	0.658	0.871	0.969

**Table 6:** Gene-specific DNA markers linked to BB resistance genes which were used in screening and selection of pyramided lines.

Gene	Chr No.	Marker Name	Primer Sequences		Expected Size (bp)	References
*Xa4*	11	MP1	Forward	ATCGATCGATCTTCACGAGG	150	[69]
		MP2	Reverse	TGCTATAAAAGGCATTCGGG		
*xa5*	5	*xa5*	Forward	GCTCGCCATTCAAGTTCTTGAG	198	[70]
			Reverse	CCTTGATAGAAACCT TGCCTTGAC		
*xa13*	8	*xa13* prom	Forward	CCTGATATGTGAGGTAGT	500	[22]
			Reverse	GAGAAAGGCTTAAGTGC		
*Xa21*	11	pTA248	Forward	CGATCGGTATAACAGCAAAAC	1000	[71]
			Reverse	AGACGCGGTAATCGAAAGATGAAA		

## Data Availability

Most of the data are available in all Tables and Figures of the manuscripts.
